# Use of mHealth Solutions for Improving Access to Adolescents' Sexual and Reproductive Health Services in Resource-Limited Settings: Lessons From Zimbabwe

**DOI:** 10.3389/frph.2021.656351

**Published:** 2021-09-28

**Authors:** Dominica Dhakwa, Fungai H. Mudzengerere, Mulamuli Mpofu, Emmanuel Tachiwenyika, Florence Mudokwani, Blessing Ncube, Mutsa Pfupajena, Tendai Nyagura, Getrude Ncube, Taurayi A. Tafuma

**Affiliations:** ^1^Family Health International 360, Harare, Zimbabwe; ^2^Zimbabwe Health Interventions, Harare, Zimbabwe; ^3^Family Health International 360, Gaborone, Botswana; ^4^United States Agency for International Development Zimbabwe Mission, Harare, Zimbabwe; ^5^AIDS and TB Department, Ministry of Health and Child Care, Harare, Zimbabwe

**Keywords:** mHealth, referral, AGYW, services, dreams, community

## Abstract

**Background:** Gaps still exist in reducing new HIV infections among adolescent girls and young women (AGYW) aged 10–24 years. High Internet coverage and mobile phone penetration rates present opportunities for the use of mobile health (mHealth) to support access to health services. We present results of an FHI 360 and Zimbabwe Health Interventions-implemented mHealth intervention for reproductive health (RH) and HIV testing service (HTS) referral among AGYW aged 10–19 years between October 2019 and September 2020.

**Methods:** Adolescent girls and young women referred for RH and HTS under the Determined, Resilient, Empowered, AIDS-free, Mentored, and Safe (DREAMS) program had automatic reminders sent to their phones to facilitate access to services through short message service (SMS) and also using a paper-based system. These data were captured in a web-based District Health Information System (DHIS) database, which captured the referral completion status of the AGYW. Data for AGYW referred for RH and HTS for the period October 2018 to September 2019 for the paper-based system and October 2018 to September 2020 for the mHealth were extracted from District Health Information System version 2 (DHIS2) database and analyzed using SPSS to generate descriptive statistics. The Chi-square test was used to assess differences in referral completion rates by age-group; marital status, district, and type of service, as well as differences between mHealth and paper-based referral completion rates within each of the groups for the variables above.

**Results:** A total of 8,800 AGYW referred for RH and HTS, where 4,355 and 4,445 were referred through the mHealth and paper-based systems, respectively. About 95.2% (4,148/4,355) and 87.8% (3,903/4,445) referred through mHealth and the paper-based system, respectively completed referrals. The median time for referral completion was 1 day (Range = 0–9 days) for mHealth and 11 days (Range = 0–28 days) for the paper-based system. AGYW referred through mHealth were 17.995 times

more likely to complete the referral system than those referred through the paper-based system (OR =17.995; *p* <0.001).

**Conclusion:** Compared to the paper-based referral system the mHealth solution resulted in a higher, service referral completion rates and shorter turnaround time. We recommend expansion of the mHealth solution to all DREAMS supported districts to increase uptake of RH and HTS among AGYW aged 10–19 years.

## Introduction

Despite reductions in HIV infections globally, gaps still exist in addressing the HIV pandemic among adolescent girls and young women (AGYW) (1). Thus, innovations like promoting a complete service referral network (SRN) through mobile health (mHealth) are critical for any HIV programming among this vulnerable group. mHealth is the use of mobile and wireless technologies to support health interventions, and literature has shown that mHealth interventions have demonstrable efficiency in supporting completion of patient referral ensuring treatment adherence especially among the youths (2). Community-based strategies like mHealth can help accelerate progress toward HIV epidemic control (3, 4). The use of mHealth through mobile devices like smartphones and tablets provides interactive programs and interventions are appealing to the young generations (5). The rise in the use of technology by youths globally presents greater opportunities for mHealth. Over 80% of youths aged between 18–29 years are believed to own a phone in developed countries, while in low and middle-income countries, the use of the Internet and owning a smartphone are also on the rise (6, 7). This makes mHealth a powerful tool capable of transforming the lives of the youths to meet their reproductive health (RH) and HIV testing service (HTS) needs. In Sub-Sahara Africa, short message service (SMS) has been used for reminding clients to undergo HIV testing and to pick up their medicine refills for those on antiretroviral therapy (ART) (8). Additionally, mHealth reduces documentation errors, is efficient in reducing the turnaround time, gives quality data, and is effective in reducing the missed appointments (9).

Family Health International (FHI360) and Zimbabwe Health Interventions (ZHI) with funding from the United States Agency for International Development (USAID) have been implementing the Determined, Resilient, Empowered, AIDS-free, Mentored, and Safe (DREAMS) program in Zimbabwe since 2015. The program aims to reduce HIV incidence among 10–19 year-old AGYW. Under the Zimbabwe DREAMS project, referrals for services were done using a paper-based system where out of school club facilitators (OOSCFs) generated referral slips which the AGYW handed over to the service providers upon presenting themselves for services. The referral slips were completed in duplicate where AGYW were given one of the slips which the service providers signed as confirmation for referral completion and services rendered on presentation. Tracking referral completions was done through the collection and reconciliation of the slips between the referral organization and the service provider. This referral system had the following challenges: poor communication between service providers, loss of referral slips, lack of feedback from the service providers, and long turnaround time for completion of the referral cycle to access services (10, 11). Under the project, mHealth was introduced on October 1, 2019. The community OOSCFs screen AGWY for eligibility into the DREAMS programs, and those who are eligible are enrolled, and their demographic details are captured into District Health Information System version 2 (DHIS2). AGYW are referred for HTS and RH services through the generation of a referral slip, which is also captured in DHIS2. The AGYW's contact telephone number is linked to the DHIS database where reminders are sent for services not accessed. Once services are accessed, information from the service providers is updated into DHIS2. This study assessed the effectiveness of mHealth in improving service linkages among AGYW. It also evaluates the turnaround time to referral completion among AGYW referred through the mHealth and the paper-based referral systems. In addition, the study explored factors associated with services referral completion among AGYW.

## Methodology

This analysis used aggregate and routine program data, extracted from the DHIS2 project database. Routine program data of AGYW enrolled in the DREAMS program and referred for RH and HIV prevention services for the period October 2018–September 2020 were extracted by the data capture clerk from DHIS2. We extracted aggregate data on the following data elements: age, marital status, district, referred health facility, date of referral, and referral outcome.

### Determined, Resilient, Empowered, AIDS-Free, Mentored, and Safe Program

Zimbabwe has been implementing the DREAMS program since 2015. The program is aimed at reducing HIV incidence among AGYW aged 10–24 years old. In Zimbabwe, the program is being implemented in six districts, namely Bulawayo, Chipinge, Gweru, Makoni, Mazowe, and Mutare. The DREAMS core package combines evidence-based approaches that go beyond health, but addresses the structural drivers that directly and indirectly increase girls' risk for contracting HIV. These include poverty, gender inequality, sexual violence, and lack of education. Thus, the program offers in-school and community-based service delivery to build social assets for the AGYW. The program also promotes changing harmful community norms that put AGYW at risk of gender-based violence (GBV). Through layering of services, the DREAMS program delivers clinical and post-GBV services. The clinical services that are delivered through the DREAMS program include HIV testing, Sexually Transmitted Infections (STI) screening, Pre-exposure prophylaxis (PrEP), Post-Exposure prophylaxis (PEP), and reproductive health.

### Enrolment of AGYW Into the DREAMS Program

The OOSCFs, who are community cadres employed under the DREAMS program, identified AGYW aged between 10 and 19 years who are at risk of contracting HIV. Using the enrolment standard operating procedures (SOPs), written consent was sought from AGYW prior to the administration of the DREAMS eligibility screening tool. The DREAMS eligibility screening tool captures demographic details, orphanhood status, indulgence in sex, experience of any GBV, alcohol misuse, and if the AGYW had experienced any of this, then they are eligible for enrollment into the program and hence referred for services. If the AGYW are eligible, a unique identification code is generated, which will be used for enrolment into the program. The DREAMS program enrolment tool was administered, which captured the demographic and social details, exposure to GBV and HIV risk assessment. Provision or referral for appropriate services was conducted based on AGYW vulnerabilities. Data on screening and enrolment of the beneficiaries were captured in the program database (DHIS2) by Monitoring and Evaluation (M&E) staff.

### Referral Process for Services

Adolescent girls and young women identified from the community who were screened and enrolled into the DREAMS program and deemed at risk of contracting HIV were either offered services onsite or referred to another implementing partner for services. A referral slip was generated and given to the AGYW, with the duplicate copy remained with the OOSCF for tracking purposes. Upon accessing services at the referral site, the referral slips were collected by a health worker who offered HTS or RH services. The issuing partner will then follow up to determine completion of referral with the service providers. Data on referral slips were later captured in DHIS2 where reminders for those who have not accessed services were generated, and notifications sent through SMS.

Since October 2019, referrals were made through the mHealth platform where slips generated manually were immediately captured into DHIS2 upon being issued to the AGYW. If services were not accessed, continuous and automated SMS reminders were sent until the AGYW access the service and an update is made in DHIS2. Referrals are made to both the public and private service providers. Among the public service providers are the Ministry of Health and Childcare (MOHCC) and Zimbabwe National Family Planning Council (ZNFPC) run facilities. Private sector service providers are mainly non-governmental organizations who conduct outreach activities.

### Sampling and Data Collection

All AGYW who were enrolled and referred for HTS and RH services in the six districts using the paper-based referral system from October 1, 2018 to September 30, 2019, and those referred through mHealth from October 1, 2019 to September 30, 2020 were included in the analysis. Data were extracted from the project's DHIS2 system.

### Data Analysis

Data were extracted from the DHIS2 database, exported to Microsoft Excel spreadsheet, and analyzed using SPSS (IBM SPSS Statistics for Windows, version 22 (IBM Corp., Armonk, NY, USA) for generating descriptive statistics. The Chi-square-test was used to test if there were differences in the completion of referrals within the following variable groups: age-group, marital status, district, service, organization referred to, and location of service within both the mHealth and the paper-based referral systems. We also used the Chi-square to assess for differences in the referral completion rates between mHealth and paper-based referral systems within each sub-group of the variables listed above. Logistics regression was used to estimate the factors associated with service completion. Cox Proportional Hazard analysis was used to test the difference in time to completion of service referral between the two systems (mHealth vs. paper-based system).

### Ethical Consideration

Ethics approval was obtained from the Medical Research Council of Zimbabwe (MRCZ/E/254). Routine monitoring and evaluation program data were analyzed. Personal identification information of beneficiaries was excluded from the analysis.

## Results

A total of 8,800 AGYW aged 10–19 years were referred for HTS and RH, where 4,355 were referred through mHealth and 4,445 through the paper-based system. Of these, 1,821 (20.7%) were from Bulawayo district while 10.9% (959/8,800) were from Mazowe district (Table 1). The majority 96.2% (8,66/8,800) were aged 15–19 years. Nearly two-thirds, 60.4% (5,315/ 8,800) of the AGYW were referred for RH services. About 91.4% (8,043/8,800) were single while 4.4% were married and 4.2% were divorced.

**Table 1:** mHealth and paper-based referral system results.

	**mHealth referral system**				**Paper-based referral system**			
	**No. referred**	***N*. completed referral**	**% referrals completion**	***P*-value**	**Number referred**	**Number completed referral**	**% referral completion**	***P*-value**
**Age-group**				** <0.001**				** <0.001**
10–14 yrs	77	72	93.5%		260	177	68.1%	
15–19 yrs	4,278	4,076	95.3%		4,185	3,726	89.0%	
**Total**	**4,355**	**4,148**	**95.2%**		**4,445**	**3,903**	**87.8%**	
**Marital status**				** <0.001**				** <0.001**
Single	4,145	3,941	95.1%		3,898	3,419	87.7%	
Married	164	161	98.2%		227	168	74.0%	
Divorced	46	46	100%		320	316	98.8%	
**District**				** <0.001**				** <0.001**
Bulawayo	567	529	93.3%		1,254	1,193	95.1%	
Chipinge	886	802	90.5%		680	584	85.9%	
Gweru	935	892	95.4%		738	601	81.4%	
Makoni	996	991	99.5%		297	292	98.3%	
Mazowe	590	568	96.3%		365	312	85.5%	
Mutare	381	366	96.1%		1,111	921	82.9%	
**Services referred**				** <0.001**				** <0.001**
HTS	1,593	1,532	96.2%		1,893	1,759	92.9%	
RH	2,762	2,616	94.7%		2,552	2.144	84.0%	
**Organization referred to**				** <0.001**				** <0.001**
MOHCC	2,731	2,638	96.6%		1,036	598	57.7%	
PSI	1,036	944	91.1%		344	341	99.1%	
ZNFPC	518	498	96.1%		2,963	2,908	98.1%	
CeSHHAR	70	68	97.1%		102	56	54.9%	
**In-school or community**				** <0.001**				** <0.001**
In-school	2,578	2,484	96.4%		1,231	1,069	86.8%	
Community	1,777	1,664	93.6%		3,214	2,832	88.2%	

From Table 1, there was a significant difference in referral completion by age-group, marital status, district, and organization for referrals done by both mHealth and the paper-based system (*p* <0.001).

Less than half, 42.8% (3,766/8,800) of the AGYW were referred to the MOHCC for HTS and RH while the rest were referred to private organizations.

### Services Referral Completion

About 95.2% (4,148/4,355) of AGYW was referred through mHealth, which was significantly higher than the 87.8% (3,903/4,445) of the AGYW referred through the paper-based system (*p* <0.001; Table 2).

**Table 2:** Differences in referral completion between mhealth and the paper-based system.

	**mHealth**	**Paper-based**	***X^**2**^***	***P***
**Age-group**				
10–14 yrs	93.5%	68.1%	19.911	<0.001
15–19 yrs	95.3%	89.0%	114.616	<0.001
**Total**	**95.2%**	**87.8%**		** <0.001**
**Marital status**				
Single	95.1%	87.7%	140.294	<0.001
Married	98.2%	74.0%	41.660	<0.001
Divorced	100%	98.8%	0.581	0.446
**District**				
Bulawayo	93.3%	95.1%	2.564	0.109
Chipinge	90.5%	85.9%	8.131	0.004
Gweru	95.4%	81.4%	83.772	<0.001
Makoni	99.5%	98.3%	4.162	0.041
Mazowe	96.3%	85.5%	36.290	<0.001
Mutare	96.1%	82.9%	41.486	<0.001
**Services referred**				
HTS	96.2%	92.9%	17.296	<0.001
RH	94.7%	84.0%	162.665	<0.001
**Organization referred to**				
MOHCC	96.6%	57.7%	937.265	<0.001
PSI	91.1%	99.1%	25.837	<0.001
ZNFPC	96.1%	98.1%	8.406	0.004
CeSHHAR	97.1%	54.9%	36.815	<0.001
**In-school or community**				
In-school	96.4%	86.8%	120.289	<0.001
Community	93.6%	88.2%	38.381	<0.001

Within all age-groups, type of service, and some districts, referral completion was higher within the mHealth referral system compared to the paper-based system (*p* < 0.001). Though referral completion was higher in the mHealth system among the divorced and those in Bulawayo district, the difference was not statistically significant (*p* = 0.446 and *p* = 0.109, respectively).

### Time to Service Referral Completion

**Figure 1:**
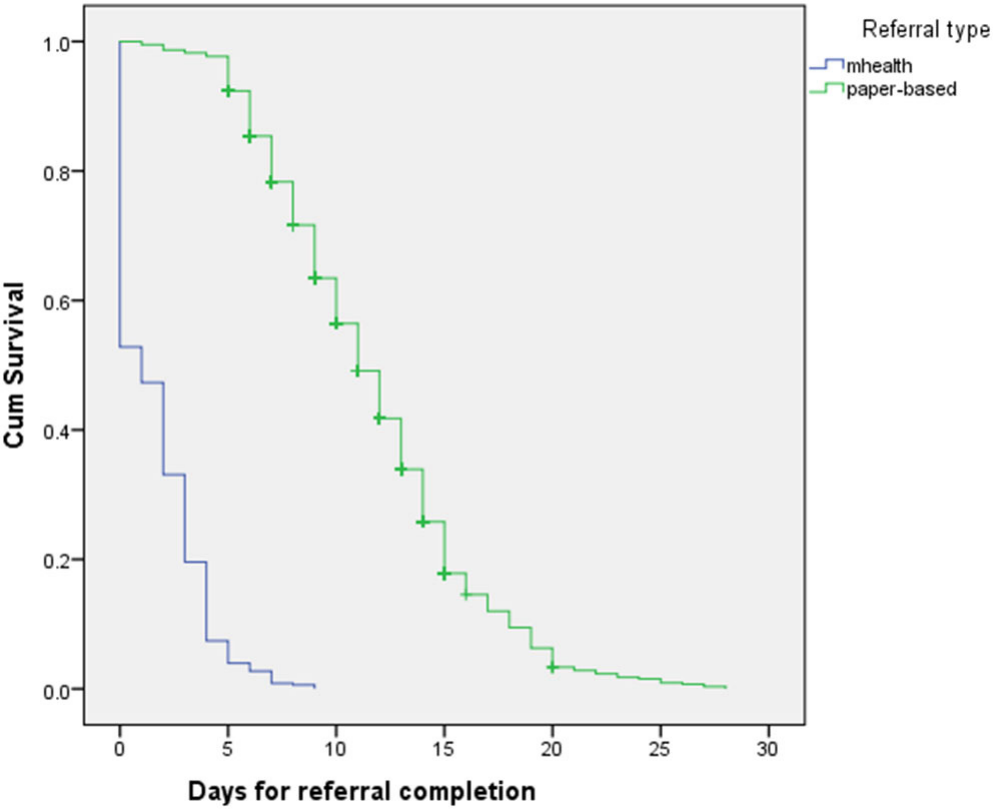
Time to referral completion for mHealth and paper-based system.

Almost half 47.2% (1,957/4,148) of the AGYW referred through the mHealth platform completed the service referral on the same day, compared to 0.8% (36/4,398) for the paper-based system (Figure 1). This rises to 96.1% (3,984/4,148) of the AGYW referred through the mHealth platform within 5 days compared to 20.7% (912/4,398) for those referred through the paper-based system.

The median time for referral completion was 1 day, (range = 0–9 days) for mHealth while it was 11 days (range = 0–28 days) for paper-based system. There was a significant difference in the time to completion service referral network for mHealth and the paper-based systems (*X*^2^ = 5868.786, *p* < 0.001). Also, the Cox regression analysis showed a significant difference in the time to completion of the referral network between mHealth and paper-based systems, Exp (*B*) = 12.157, 95% CI (11.308; 13.070).

### Factors Associated With Service Referral Completion

Adolescent girls and young women referred through mHealth were 17.995 times more likely to complete referral than those referred through the paper-based system [OR = 17.995 (95% CI =13.520: 23.952)]. AGYW aged 10–14 years old were 75.2% less likely to complete service referral than those aged 15–19 years [OR = 0.248; 95% CI (0.169; 0.363)]. Single AGYW were also less likely to complete SRN than the married and the divorced [OR = 0.137; 95% CI (0,049; 0.381); *p* < 0.001].

## Discussion

Results from this study showed high referral completion rates among AGYW referred using mHealth than those referred using a paper-based system. mHealth services that integrate SMS reminders help to remind AGYW to access services unlike referral slips which can easily be misplaced. The high mobile phone penetration rate among AGYW also assured that enrolled clients will receive SMS reminders. While the use of mHealth was successful within DREAMS in Zimbabwe, this contrasts findings from a study conducted in Uganda, where the use of mHealth had little effect in improving access and completion of services referred due to limited data bundles and support systems for online follow-up and reporting (12). In Zimbabwe, the use of mHealth timeously reminded AGYW to seek services early, and these results are similar to findings from studies conducted in other low-income settings (13). While mHealth has benefits as shown in this study, a similar study in 2016 found that the use of mHealth had a cost implication as the beneficiaries had challenges of mobile data to access the system, which was resolved by the use of SMS reminders that were cost-effective (14). The use of SMS to remind AGYW improved accessing services and reduced the referral completion turnaround time, similar to the findings from studies from other African countries (8, 15).

There were high HTS and RH service referral completion rates regardless of the marital status of the AGYW, which can be attributed to the integration of HTS and RH for AGYW as reported from other Sub-Saharan countries (16). There was high service uptake among AGYW enrolled in the in-school program compared to the out of school program. The DREAMS program through the in-school activities equips AGYW with knowledge and refers them for services. In Kenya and South Africa, in-school AGYW were to have a lower vulnerability (17, 18).

Most of the AGYW referred using the mHealth innovation accessed HTS and RH services within 5 days, compared to less than a quarter among those referred using the paper-based system. Those enrolled into the mHealth platform received constant reminders, which could have prompted service uptake. Moreover, availability of service directories, which are booklets with names and contact details of service providers, and the use of mHealth in tracking and following clients equipped the AGYW with the tools to access services. The study findings are consistent with the findings for other health interventions from South Africa, where mHealth was preferred among AGYW because it is user-friendly, appeals to youth, and has led to short turnaround times for accessing family planning and HIV self-test services in South Africa (19, 20).

### Limitations

This study has limitations. Completion of service referrals is based on data on referrals captured in DHIS, and some referral slips could have been lost before capture. The analysis was limited to AGYW enrolled into the DREAMS project covering only six districts in a country with more than 57 districts, which makes it difficult to generalize findings beyond study districts. In addition, the analysis used routinely collected program data thereby limiting analysis to variables captured in the database.

### Conclusion and Recommendations

The study highlights the utility of mHealth in increasing the uptake of HTS and RH services among AGYW. The use of mHealth was effective in reducing the turnaround time from the time of referral to completion among AGYW referred for HTS and RH services and should be expanded to other districts and also to other health services targeting especially youth.

## Data Availability Statement

The original contributions generated for in the study are included in the article/Supplementary Material, further inquiries can be directed to the corresponding author.

## Ethics Statement

The studies involving human participants were reviewed and approved by Medical Research Council of Zimbabwe. Written informed consent to participate in this study was provided by the participants' legal guardian/next of kin.

## Author Contributions

DD, BN, and FM development of the manuscript. TT and GN review of manuscript. ET manuscript development and review. MM data analysis and review of manuscript. TN manuscript development. MP development of manuscript and review. FHM development of manuscript, data analysis, and review of manuscript. All authors contributed to the article and approved the submitted version.

## Conflict of Interest

The authors declare that the research was conducted in the absence of any commercial or financial relationships that could be construed as a potential conflict of interest.

## Publisher's Note

All claims expressed in this article are solely those of the authors and do not necessarily represent those of their affiliated organizations, or those of the publisher, the editors and the reviewers. Any product that may be evaluated in this article, or claim that may be made by its manufacturer, is not guaranteed or endorsed by the publisher.

## Abbreviations

AGYW: Adolescent Girls and Young women
AIDS: Acquired Immunodeficiency Syndrome
ART: Antiretroviral Therapy
CeSHHAR: Center for Sexual Health and HIV and AIDS Research
DREAMS: Determined Resilient Empowered AIDS-Free Mentored and Safe
DHIS2: District Health Information System version 2
FHI360: Family Health International
FP: Family Planning
GBV: Gender-based Violence
HIV: Human Immunodeficiency Virus
HTS: HIV Testing Services
IQR: Interquartile Range
mHealth: Mobile health
MOHCC: Ministry of Health and Child Care
MRCZ: Medical Research Council of Zimbabwe
NGO: Non-governmental Organizations
OOSCF: Out of School Club Facilitators
PEP: Post-Exposure Prophylaxis
PrEP: Pre-Exposure Prophylaxis
PSI: Population Services International
RH: Reproductive Health
SMS: Short Messages Services
SRN: Service Referral Network
SPSS: Statistical Package for the Social Sciences
STI: Sexually Transmitted Infection
TB: Tuberculosis
USAID: United States Agency for International Development
ZHI: Zimbabwe Health Interventions
ZNFPC: Zimbabwe National Family Planning Council.
